# Pathological Characteristics of Triple-Negative Breast Cancer at Main Referral Teaching Hospital, April 2014 to April 2015, Tehran, Iran

**Published:** 2016-10-01

**Authors:** Alireza Abdollahi, Mehrnoosh Etemadi

**Affiliations:** 1 Associate Professor of Pathology, Department of Pathology, Imam Hospital Complex, Tehran University of Medical Sciences, Tehran, Iran; 2 Students of Scientific Research Centre, Tehran University of Medical Sciences, Tehran, Iran

**Keywords:** Triple-negative breast cancer, Epidemiology, Iranian patients

## Abstract

**Background** **:** Triple-negative breast cancers (TNBC) are defined as breast cancers with lack of estrogen and progesterone receptors and no overexpression of human epidermal growth factor receptor 2 (HER2). This study was performed to determine the frequency and pathologic features of TNBC in Iranian patients.

**Subjects and Methods:** This cross-sectional study was performed on patients with breast cancer who referred to Cancer Institute, affiliated to Tehran University of Medical Sciences, from April 2014 to April 2015. Data about the demographics, the status of gene receptors and the pathologic features were extracted from patients’ records.

**Results** **:** Of 214 pathology samples of patients with malignant breast cancer, TNBCs account for 14% of cases. The mean age in N-TNBC group was 50 ± 12 years. Significant difference was seen between the age of two groups (p=0.03). No significant difference was observed regarding the number of involved lymph nodes between two groups (p=0.058). Presence of vascular and nerve invasion and involvement of surgical margins at the time of diagnosis were significantly more frequent in TNBC group comparing with N-TNBC. Grade III of histologic and nuclear grading was significantly more common in TNBC.

**Conclusion** **:** TNBC group was significantly associated with higher grade, higher mitotic indices and higher rate of P53 positivity and higher level of Ki-67 at the time of diagnosis. High grade breast cancers are more seen in TNBC. The presence of aforementioned characteristics in a patient highlights the need for evaluating TNBC biomarkers to better predict prognosis and consider appropriate treatment.

## Introduction

 Breast cancer (BC) is the second common cancer worldwide, accounts for about 10.4% of all cancers. It is also the second common cause of cancer death in females.^1^^-^^3^ Breast cancer is the main cause of mortality in women aged 45 to 55.^3^^-^^4^ In 2013, nearly 234,000 women were diagnosed with breast cancer in US, 39,000 of who have died.^5^ Diagnostic evaluation of breast cancers is used commonly with immunohistochemistry (IHC) staining for three biomarkers of estrogen receptor (ER), progesterone receptor (PR) and human epidermal growth factor receptor2 (HER2).^4^ One of the most challenging breast cancer types is TNBC. Triple-negative breast cancer (TNBC) is referred to a type of BC which is negative for these three mentioned biomarkers. Indeed, in TNBC patients, genes of these three biomarkers are not expressed. Despite the similarity in basic diagnosis of TNBC and other types of BC, different factors distinguish this type of cancer from other types. Some of these factors are difference in the age of patient at time of diagnosis, race, risk factors, pathologic and molecular properties, normal course of this disease, sensitivity and response to chemotherapy.^5^^,^^6^ This type of cancer often has a more aggressive nature compared with other types of breast cancer; then, routine hormonal treatments are ineffective for that.^7^ In recent years, TNBC has attracted the attention of therapeutic and counseling cancer centers in different countries.^7^ TNBC is an interesting subject for research due to the following five reasons:

1) TNBC is considered a bad prognostic actor for disease free survival and overall survival, 2) No effective treatment has so far been developed for this type of cancer, 3) This cancer is further seen in women of pre-menopause age and of African race, 4) There is a significant overlapping between Basal like phonotype and TNBC and 5) There is a significant overlapping between TNBC and BRCA1 enriched breast cancers.^8^

TNBC is often diagnosed with a high-grade ductal histology and the increased amount of mitosis and cell proliferation.^9^ Due to lack of hormone receptors and HER2 expression and subsequently, lack of response to hormone treatments and Transtuzumab, there is weak early warning sign.^9^ Compared with other subtypes, metastasis to viscera particularly lung and brain and to bones is less common.^10^ Furthermore, this subtype has a low survival and high relapse particularly over three to five years following diagnosis.^9^ No standard treatment regimen has been registered for TNBC and also, there is insufficient information available to that affect. Although this type of cancer is initially sensitive to chemotherapy, it is significantly more invasive than other tumors.^11^ Regarding the high prevalence of BC in Iranian females, geographic variation in distribution and clinic-pathological specifications of this cancer, this study aims to determine demographics and histopathologic features of this type of breast cancer (TNBC) in Iran and then, comparing that with non-TNBC (N-TNBC).

## SUBJECTS AND METHODS

**Study population**

This cross-sectional study was performed on patients with breast cancer, who referred to Cancer Institute, affiliated to Tehran University of Medical Sciences from April 2014 to April 2015.

All pathology sample records of patients with breast mass which were diagnosed as a malignant breast tumor were included in this study. Samples with absence or incomplete immunohistochemistry report for the respective pathology were excluded.

Immunohistochemically stained slides were evaluated for the presence of positive reaction, cellular localization (nuclear or cytoplasmic), pattern of staining (focal or diffuse) and intensity of reaction in individual tumor cells (strong or weak). Any positive nuclear reaction for ER and PR, irrespective of percentage of reactive cells, was recorded as positive.

The IHC test gives a score of 0 to 3+ that measures the amount of HER2 receptor protein on surface of cells in a breast cancer tissue sample. If the score is 0 to 1+, it is called “HER2 negative” (No or weak staining). Incomplete membrane staining in any % of cells is defined as score 1+. If the score is 2+, it is called "borderline, Equivocal" (Strong complete homogeneous membrane staining (chicken wire pattern) in ≤30% of cells or weak/moderate heterogeneous complete membrane staining in at least 10% of cells).

A score of 3+ is called “HER2 positive” (Strong complete homogeneous membrane staining (chicken wire pattern) in >30% of cells). Ki-67 is a nuclear non-histone protein that is present at low levels in quiescent cells but is increased in proliferating cells, especially in the G2, M and latter half of the S phase. Only nuclear staining (plus mitotic figures which are stained by Ki-67) should be incorporated into the Ki-67 score that is defined as the percentage of positively stained cells among total number of malignant cells scored. Scoring should involve the counting of at least 500 malignant invasive cells (and preferably at least 1000 cells).

The ethics committee of Tehran University of Medical Sciences approved the study protocol according to the declaration of Helsinki.

Malignant breast tumors with negative status for ER, PR, HER2 and IHC biomarkers were defined as TNBC group and were considered as case group. Other patterns which were N-TNBC group which were considered as control group. According to pathology reports, frequency, age, sex, cancer type, tumor size, tumor grade, tumor location, the benign accompanied lesion, presence of lymph node involvement and the number of involved lymph nodes, presence or absence of in situ component, skin involvement, nipple involvement, involvement of the surgical margins, vascular invasion or perineural invasion, mitoses, Ki-67 proliferative factor percent, necrosis, nuclear grade, calcification and granulomatous reaction were compared between two groups.

**Data analysis**

The statistical package of social science, version 19.0 (SPSS, Chicago, Illinois, USA) was used for data analysis. Statistical significance was noted for p≤0.05. For finding the association between qualitative variables, Chi-Square test was used, while independent sample t-test and ANOVA test were applied for comparison of quantitative variables.

## Results

 Two hundred and fourteen pathology samples of patients with breast cancer were evaluated. Thirty patients (14%) were negative for all three receptors (TNBC group) and 184 patients (86%) belonged to N-TNBC group. The mean age of patients in TNBC group was 43 ± 12 years (26 to 85 years). The mean age in N-TNBC group was 50 ± 12 years (24 to 91 years). Significant difference was seen between the age of two groups (p=0.03). One hundred and eighty one of the patients (98.4%) were female and 3 patients (1.6%) were male in N-TNBC group. TNBC group were all females. Significant sex difference was not seen between two groups (p=0.48).

Tumor size in the TNBC group was 3.83 ± 1.88 within the 1-10 cm range and was 2.98 ± 2.22 in N-TNBC group, within the 0.2-13 cm range. Although the mean size of tumor is greater in TNBC group, there was no significant differences regarding the tumor size between two groups (p=0.72). The number of involved lymph nodes in TNBC group was 3 ± 3 within the 0-22 range and in N-TNBC was 2 ± 2 within the 0-7 range. No significant difference was observed regarding the number of involved lymph nodes between two groups (p=0.058). TNBC group was significantly associated with younger age, higher grade, higher mitotic indices and higher rate of P53 positivity and higher level of Ki-67 at the time of diagnosis. Presence of vascular and nerve invasion and involvement of surgical margins at the time of diagnosis were significantly more frequent in TNBC group comparing with N-TNBC.

Table 1 shows other pathologic specifications of breast tumor in these two groups. Grade III of cellular and nuclear grading as well as involvement of left lower quadrant (LLQ) was significantly more common in TNBC. The microscopic and macroscopic characteristics of breast tumor of two groups are compared in Table 2.

**Table 1:** Pathologic characteristics of Breast cancer in case and control groups

	**TNBC ** **(n=30)**	**N-TNBC ** **(n=184)**	**p-value**
**Number of mitosis**	3.60	2.90	0.01
**P53 (N/P)**	11/19	123/61	0.002
**Ki-67**	0.16	0.18	0.25
**Necrosis (N/P)**	20/10	136/48	0.40
**Vascular invasion (N/P)**	18/12	119/65	0.04
**Calcification (N/P)**	22/8	154/30	0.16
**Nerve invasion (N/P)**	23/7	163/21	0.03
**Nipple involvement (N/P)**	29/1	170/14	0.39
**In situ component (N/P)**	19/11	118/66	0.93
**Associated benign tumor (N/P)**	24/6	150/34	0.84
**Involvement of Margins (N/P)**	17/13	161/23	0.001
**Granulomatosis reaction (N/P)**	30/0	180/4	0.41
**Skin involvement (N/P)**	27/3	170/14	0.65

**TNBC:** Triple-negative breast cancer, **N-TNBC:** Non-triple-negative breast cancer

## Discussion

 In present study, the prevalence of TNBC among Iranian patients with breast cancer found to be 14%. TNBC is associated with younger age, higher grade, higher mitotic indices, higher P53 positivity and higher level of Ki-67 at the time of diagnosis. Besides, vascular and nerve invasion and invasion to surgical margins were more frequently seen in TNBC compared with N-TNBC tumors. The in situ components of both ductal and lobular types were significantly more present in TNBC but it was true for neither invasive ductal carcinoma nor invasive lobular carcinoma.

**Table 2:** Microscopic and macroscopic characters of tumor in case and control groups

**Series**	**Variable**	**TNBC**	**N-TNBC**	**p-value**
**1**	**Tumor type**			
	**IDC or ILC**	27 (90%)	167 (90.8)	0.80
	**DCIS**	3 (10%)	6 (3.3%)	0.04
	**LCIS**	-	10 (5.4%)	0.001
	**MC**	-	1 (0.5%)	-
**2**	**Tumor histologic grading**			
	**I**	3 (10%)	15 (8.2%)	0.87
	**II**	26 (86.7%)	152 (82.6%)	0.66
	**III**	1 (3.3%)	17 (9.2%)	0.02
**3**	**Nuclear grading**			
	**I**	4 (13.3%)	14 (7.6%)	0.05
	**II**	24 (80%)	147 (79.9%)	0.91
	**III**	2 (6.7%)	23 (12.5%)	0.01
**4**	**Tumor side**			
	**Right**	17 (56.7%)	108 (58.7%)	0.79
	**Left**	13 (43.3%)	75 (40.8%)	0.65
	**Bilateral**	-	1 (0.5%)	-
**5**	**Tumor location**			
	**RUQ**	21 (70%)	108 (58.7%)	0.09
	**RLQ**	3 (10%)	25 (13.6%)	0.76
	**LUQ**	3 (10%)	16 (8.7%)	0.87
	**LLQ**	1 (3.3%)	26 (14.1%)	0.01
	**Subareol**	2 (6.7%)	8 (4.3%)	0.06
	**All sides**	-	1 (0.5%)	-

**TNBC:** Triple-negative breast cancer, **N-TNBC:** Non-triple-negative breast cancer, **DCIS: **Ductal carcinoma in situ, **LCIS:** Lobular carcinoma in situ, **IDC:** Invasive (or infiltrating) ductal carcinoma, **ILC:** Invasive (or infiltrating) lobular carcinoma, **MC:** Medullary carcinoma, **RUQ:** Right upper quadrant, **RLQ:** Right upper quadrant, **LUQ:** Left upper quadrant, **LLQ: **Left lower quadrant

In study performed by Dent et al. in Canada, clinical characteristics, history, recurrence pattern and the course of disease in women with TNBC was compared with other types of breast cancer. This cohort study was done on 1601 patients who were diagnosed with breast cancer in Toronto hospitals, from January to December 1987. TNBC was diagnosed in 11.2% of patients. Comparing TNBC to others, the mortality of TNBC was higher in first 5 years after the diagnosis. The maximum risk of remote metastasis in TNBC was in the first 3 years after diagnosis; afterward this risk was rapidly declined. While in other groups, this risk was almost stable during the time. This study showed that TNBC has a more aggressive clinical course.^8^

In 2008, Liedtke et al. performed a cohort study on 255 patients with TNBC and their response to neoadjuvant chemotherapy was evaluated. According to this research, higher relapse rate in visceral organs and soft tissue was seen in patients with TNBC while they had lesser bone involvement compared to other types. Moreover, survival rate after relapse time was lower in TNBC patients. Pathologic complete response rate (PCR) was higher in TNBC and this group had a good survival after neoadjuvant chemotherapy. However, if the patients remained with residual disease after neoadjuvant chemotherapy and were diagnosed as TNBC type, the prognosis was very poor.^9^ In the same year, Lin et al. in United States, performed a study with the purpose of defining metastasis risk and determining disease course in metastatic TNBC patients including those with central nervous system (CNS) metastasis. From January 2000 to June 2006, 116 patients were selected by obtaining their pathology and drug history in Dana-Farber cancer institute. According to this study, from the time which metastasis was diagnosed, the mean survival time was estimated 13.3 months. Once the metastasis was diagnosed, 14% of patients had CNS involvement. Overall, in 46% of the patients, CNS metastasis was diagnosed before death time. Mean survival time after diagnosing CNS metastasis, was 4.9 months. By excluding race and age effect, mortality in patients whom CNS involvement was their first sign, considered 3.4 times higher than other patients with TNBC. Results of this study showed that the survival of TNBC after relapsing is poor and new therapeutic strategies are needed. Also, it was revealed that high rates of CNS involvement are not seen in TNBC.^10^ In addition, Anders et al. in North Carolina, performed a cross-sectional study to evaluate the age, race, subtype and prognosis of breast cancer patients with brain metastasis. According to this survey, while investigating brain relapses, extra-cranial metastasis was diagnosed in 83% of the patients, which indicates the systemic nature of this disease. Mean survival time of TNBC patients after CNS metastasis were less than six months.^11^ In 2010, a good review by Foulkes et al. reviewed etiologic factors as well as clinical and molecular characteristics and the treatment of TNBC. Based on this survey, the relapse rate of TNBC compared to other types was higher and its prognosis was poorer.^12^ De Laurentiis et al. in Italian study reviewed the current TNBC therapeutic choices. They mentioned that TNBC is sensitive to chemotherapy with anthracycline and neoadjuvant based on taxane, which should be considered as the treatment method of TNBC.^13^

At the same year, Santana-Davila et al. in Florida published an article about different available choices for TNBC treatment such as Platinum agents, anti-tubulin agents. Anti angiogenic agents and multi kinase inhibitors were studied and confirmed regimen for TNBC chemotherapy. More over neoadjuvant chemotherapy is preferred regimen in patients with TNBC but there is no preferred agent in neoadjuvant chemotherapy method.^14^

TNBC accounts for 10–20% of all breast carcinomas in different countries, based on the thresholds defined for ER and PR positivity and methods for HER2 assessment.^15^^,^^16^ The main characteristics of TNBC that have retrieved from several related studies including the fact that TNBC affects younger patients (<50 years),^8^^,^^17^ is more prevalent in African-American race^16^ and almost always presents with more aggressive course.^15^ Patients with TNBC have a significantly poorer and shorter survival after the first metastasis occurred, compared with N-TNBC types.^8^ TNBC often is diagnosed in high histological grade,^18^ but about 10% of TNBCs have been reported to be of grade I in one study.^8^ There are controversies about the prevalence of lymph node metastasis in patients with TNBC; Dent et al. reported higher prevalence of lymph node metastasis in TNBC compared with N-TNBC,^8^ but other studies did not find any difference between TNBC and N-TNBC.^17^^-^^19^ It has been reported that, unlike N-TNBC, there was no correlation between tumor size and presence of lymph node metastasis in TNBC.^8^ Among epidemiologic studies in Asia, Suresh et al. investigated the epidemiological and clinical profile of TNBC at their institute in India. Characteristic data on 171 patients with TNBC gathered from 2008 to 2010. The mean age was 49 years (22-75 years). Just eight patients (5%) had a family history of breast or ovarian cancer. One hundred and six patients (62%) were in stage II, 26 (15%) in stage III, 21 in (12%) stage I and as the less common stage, 18 patients (10%) were in stage IV.^20^

Our study was designed to look at the demographic profile and histopathologic data of TNBC in Iranian population. To our knowledge, this is possibly the largest study on TNBC done in Iran.

Our TNBC population was slightly younger (median age 43 years) than the ones described in western data (median age 53 years).^8^ The finding of younger median age most likely reflects that the general trend of breast cancers occur a decade earlier in Iran.

Amirikia et al. analyzed patients with breast cancer, from California Cancer Registry (CCR) between 1988 and 2006. In their study, white Americans identified as non-Hispanic whites (NHWs) and African Americans identified as non-Hispanic blacks (NHBs). Epidemiologic data of 375,761 invasive breast cancers were investigated (containing 276,938 in NHWs and 21,681 in NHBs). Patients from NHBs were younger than NHWs (median ages 57 years and 64 years, respectively). NHBs had higher incidence rates of stage III and stage IV and a higher incidence of TNBC in all age categories.^21^

Overall, there is emerging evidence from several epidemiological studies regarding the major characteristics of this group of breast cancer which have a relatively poorer prognosis than other breast cancer subtypes. In a recent review by Boyle et al. TNBC reported as 10%–20% of invasive breast cancers which has been shown more in younger age, deprivation status, African-American race, more advanced disease stage, higher grade, high mitotic indices, family history of breast cancer and BRCA1 mutations.^22^ This was compatible with our findings. Any size or stage of breast cancer will have poorer prognosis, if its grade is higher. So, in our study cellular and nuclear grading were both evaluated and higher cellular grading is reported in TNBC of Iranian race. Cellular grading was grade II in 82.6% of patients and the second most common was grade III (9.2%). Also, the common nuclear grading was grade II (79.9%) and the second most common was grade III (12.5%). This result indicates that high grade breast cancers are more seen in TNBC, while in N-TNBC group, grade I was in second position, in both cellular and nuclear grading.

## CONCLUSION

 The presence of aforementioned characteristics in patient highlights the need for evaluating TNBC biomarkers, to better predict the prognosis and consider appropriate treatment.
